# Two Cases of Positional Variation of the Cecum and Appendix With a Vascular Anomaly: A Diagnostic Dilemma

**DOI:** 10.7759/cureus.27091

**Published:** 2022-07-21

**Authors:** Khizer Hussain Afroze M, Sangeeta Muralidharan, Aakash Shanmugam, Abdul Wahad Khan, Shaliny Bhowmik

**Affiliations:** 1 Anatomy, MVJ Medical College and Research Hospital, Bangalore, IND

**Keywords:** vascular variations, malrotation, appendix, caecum, positional variations

## Abstract

The arrangement of the alimentary canal derived from the midgut exhibits a wide range of alterations. In this article, we report two cases in male cadavers aged 65 years and 70 years, respectively, in the Department of Anatomy, MVJ Medical College and Research Hospital, wherein we discovered the positional variation of the cecum and appendix with a vascular anomaly, thus the specimens were photographed to understand further.

The observation of the first specimen is that the cecum and appendix were found in close proximity to the inferior surface of the right lobe of the liver with a vascular anomaly. The branching pattern of the superior mesenteric artery varied from the usual pattern. The right colic artery and ileocolic artery arose as a common trunk. The cecum and appendix were identified in the right hypochondrium in the second specimen, with an unusually long appendix measuring around 22.3 cm in length with maximal breadth near the base.

Knowledge of these positional variations, as well as the coexistence of the cecum and appendix with a vascular anomaly, which could provide a diagnostic conundrum, would aid in diagnosing patients of appendicitis with unusual presentations and planning optimal incisional procedures prior to surgery.

## Introduction

The arrangement of the alimentary canal derived from the midgut exhibits a wide range of alterations. Generally, as a rule, the large intestine extends from the ileocecal valve to the anus. The cecum descends to its adult position in the right iliac fossa during the establishment of the midgut loop, with a substantial decline in the size of the right lobe of the liver, followed by sequential rotation and fixing. It has an average length of 6 cm and a width of 7.5 cm, and it is surrounded on both sides by the peritoneum [1-2].

The appendix is a tube-like diverticulum that usually emerges from the posteromedial wall of the cecum. The length and position of the appendix can vary significantly, posing a diagnostic conundrum in many cases, especially since appendicitis is a life-threatening emergency that must be treated as soon as possible [3].

The disturbance in mechanisms govern­ing these rotations and fixation may result in malrotation or positional variation of the cecum and appendix. The developmental variation of the cecum and appendix are relatively rare and little attention is paid to midgut malrotation as the cause of symptoms in adults [2]. In this article, we examined positional variations and the co-existence of the cecum and appendix with a vascular anomaly, which could pose a diagnostic dilemma and can be life-threatening if detected late.

## Case presentation

Case 1

**Figure 1:**
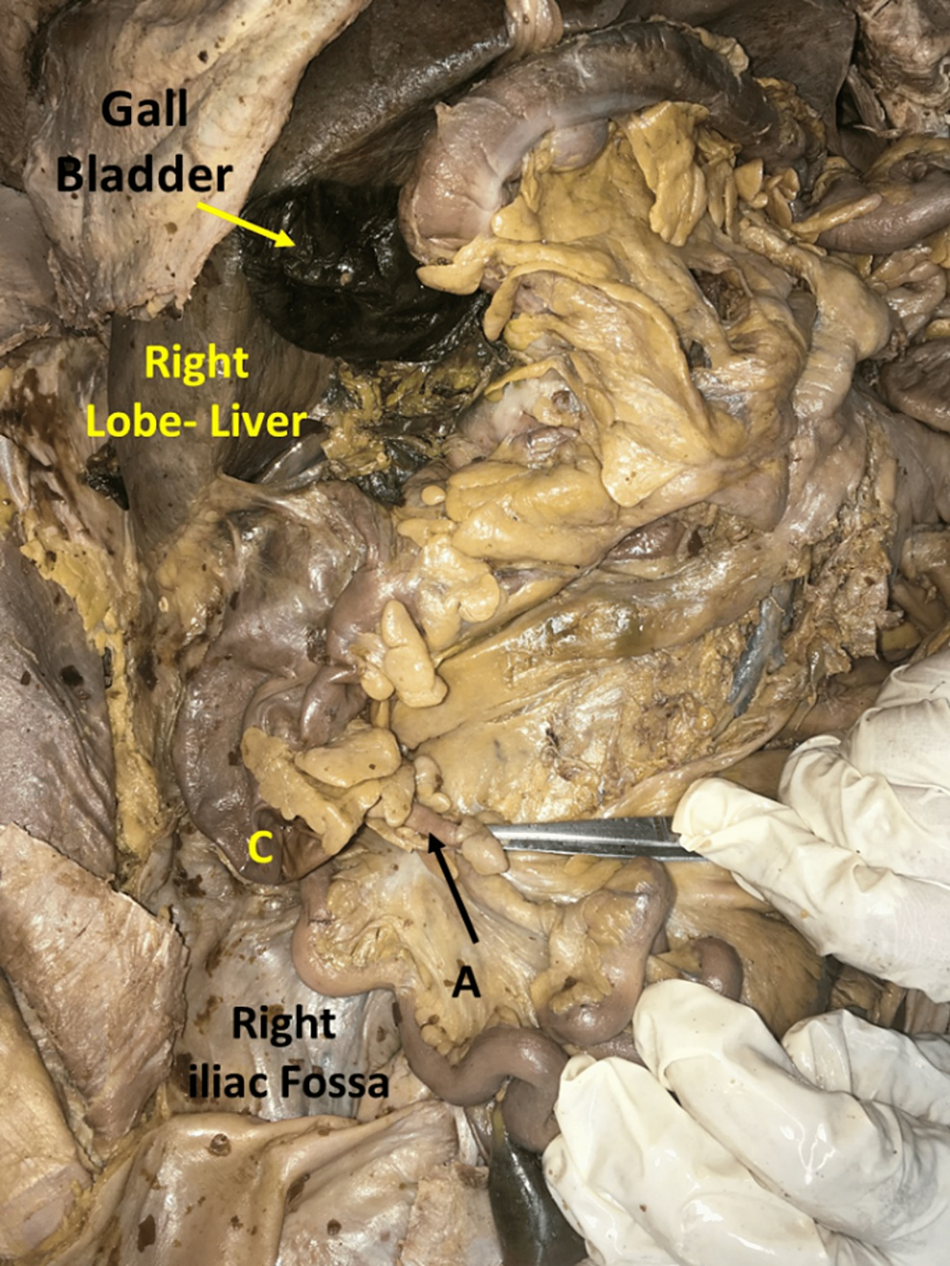
Showing the subhepatic cecum (C) with the appendix (A) in its posteromedial aspect (black arrow)

**Figure 2:**
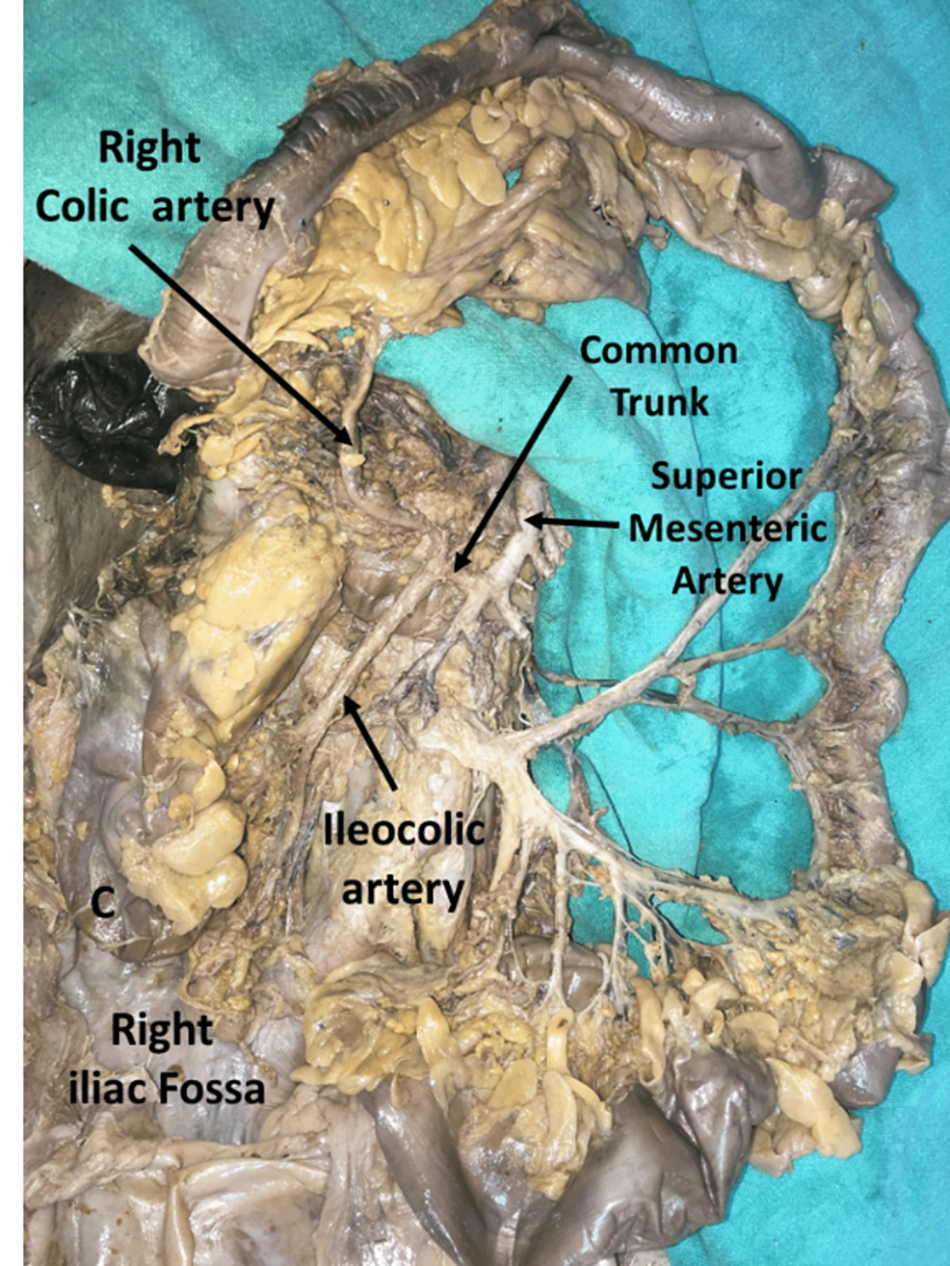
Variation in the superior mesenteric artery showing a common trunk for the right colic and ileocolic artery

During routine cadaveric dissection by first-year medical students of MVJ Medical College and Research Hospital, a variation was encountered in a male cadaver aged approximately 65 years where the cecum and appendix were found in close proximity to the inferior surface of the right lobe of the liver when compared to the original position in the right iliac fossa. In this case, the right iliac fossa was found to be empty. The cecum was quadrate in shape, the length being 7 cm and width being 8 cm, measuring from the ileocecal orifice to its lowest point. The appendix was found in the posteromedial aspect of the cecum with a pelvic (5 o'clock) position and measuring about 6 cms (Figure 1 and Figure 2).

There was also a variation in the blood supply, with the superior mesenteric artery (SMA) having a normal origin and course but a different branching pattern. The right colic artery (RCA) and the ileocolic artery (ICA) arose as a common trunk (Figure 2), which ran for a short distance of about 1.3 cm. No other variation was encountered in the branching pattern of the superior mesenteric artery.

Case 2

We observed a positional variation of the cecum and appendix with no vascular abnormality in a male cadaver aged roughly 70 years during routine cadaveric dissection of the abdominal cavity by first-year MBBS students in the Department of Anatomy at MVJ Medical College and Research Hospital.

**Figure 3:**
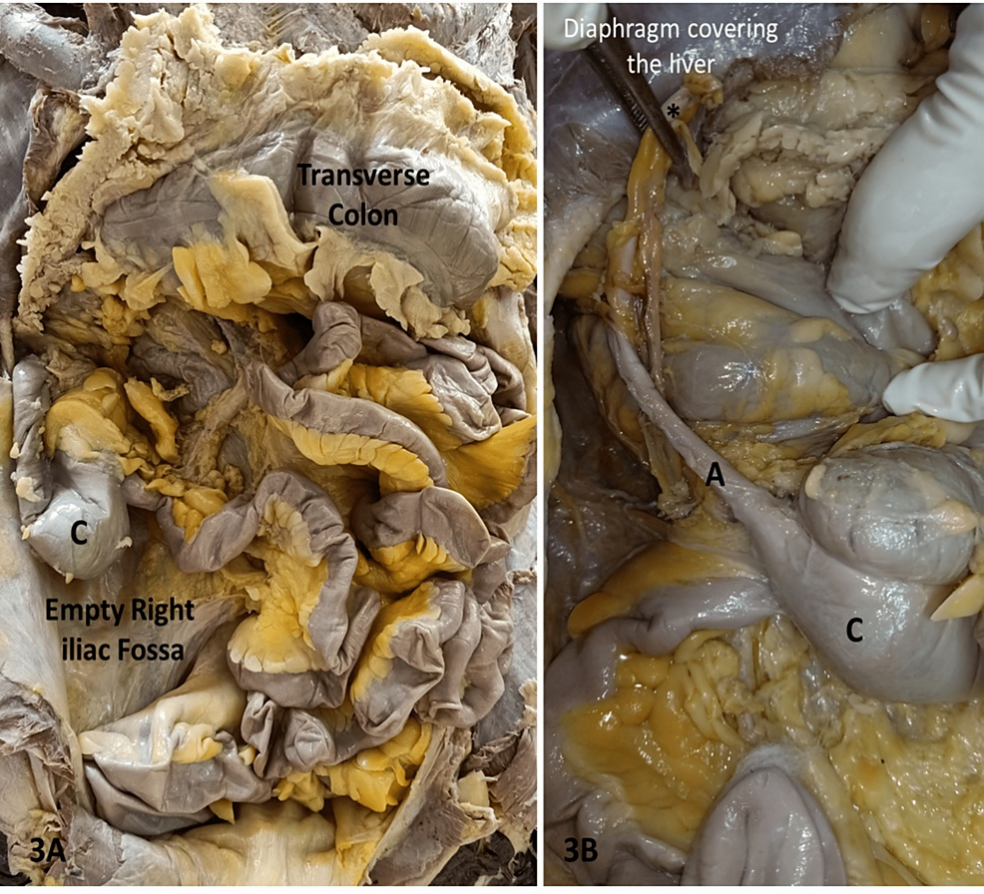
Showing the subhepatic cecum (C) 3b: shows the abnormally elongated appendix (A) with its tip (*) in contact with the liver

In the present case, the position of the cecum and appendix was found in the right hypochondrium in proximity to the inferior surface of the right lobe of the liver. The right iliac fossa was found to be completely empty. The ascending colon was found to be absent, and the cecum was found to be continuous as the transverse colon right below the liver's inferior surface. The dimension of the cecum was observed to be normal and adult type (Figure 3). 

In the retrocecal position, the appendix was revealed to be unusually lengthy, measuring about 22.3 cm with maximum breadth near the base. It was measured by using a vernier caliper. The tip of the appendix was in contact with the inferior surface of the liver, which was unique and not reported in the literature to date.

## Discussion

Intestinal rotational anomalies affect up to 1% of the total population, with 50% to 60% of instances occurring in the first four weeks of life [4].

Midgut rotation involves three stages: Stage 1 - Stage of physiological hernia during the sixth week of intrauterine life (IUL), Stage 2 - reduction of the herniated loop into the abdominal cavity around the 10th week of IUL, and Stage 3 - fixation of the gut in the abdominal cavity. During the reduction of the physiological hernia, the cecum is the last part of the post arterial segment of the midgut loop to return owing to its bulky nature. Subsequently, it descends to the right iliac fossa during the stage of fixation. As a result, an error involving the stage of fixation during midgut rotation might be a contributing factor in the development of subhepatic cecum.

Another possible explanation for the aforementioned condition is that it might also be caused by the cecum not adhering to the expanding posterior abdominal wall, or as a consequence of an intrinsic growth defect in the ascending colon, or lack of expansion in the developing or growing colon [5].

No known species of amphibian have a cecum, which may be found in most amniote species and lungfish. For most reptiles, it is generally a single midline structure that is formed as an extension of the large intestine on the dorsal side. The right iliac fossa is where the cecum and appendix are normally located in humans. It is quite common for herbivorous mammals to have a big cecum, which houses a huge number of bacteria that help in the breakdown of plant components such as cellulose. In many species, it's even larger than the colon itself [6].

Obligatory carnivores, on the other hand, who depend on meat as their major source of protein and whose diets are devoid of plant material, have a smaller cecum, which is usually replaced in part or entirely by the appendix. Raccoons, bears, and the red panda are examples of mammalian species that do not have a cecum. Anaerobic bacteria make up the vast majority of the gut flora, however, they are outnumbered by aerobic bacteria in the cecum [7].

Positional variations in the cecum and appendix can have a causative etiology and can present with different manifestations. The pelvic cecum, lumbar cecum, and subhepatic cecum are all examples of aberrant positional variations of the cecum that occur often [8]. The incidence of the subhepatic cecum is listed in Table 1.

**Table 1:** Shows the incidence of the subhepatic cecum and appendix in previous studies

Author	Year	Population	Method Of Study	No. Of Cases	Incidence
Treves et al [9]	1885	London	Cadaveric	2	Case report
Lockwood et al [10]	1892	-	Cadaveric	1	Case report
Smith et al [11]	1911	New York	Fetal Autopsy	1050	6%
Palanivelu et al [12]	2007	Indian	Laparoscopic surgery	18	0.09%
Abougabal AM et al [13]	2012	Egypt	Ultrasound + CT Scan	15	100%
Banerjee et al [14]	2012	Indian	Cadaveric	25	4%
Nagashree MV et al [4]	2013	Indian	Cadaveric	1	Case report
Das SS et al [5]	2014	Indian	Cadaveric	1	Case Report
Sehgal G et al [2]	2015	Indian	Cadaveric	1	Case Report
Chong HC et al [15]	2016	Malaysia	CT +Laparoscopic surgery	1	Case Report
Yu D et al [16]	2020	China	Ultrasound	1591	1.13%
Present Series	2021	Indian	Cadaveric	2	Case Series

The second variation observed in one of our cases, in addition to the subhepatic cecum, was a vascular variation involving the superior mesenteric artery. We encountered two classifications based on the branching pattern of SMA proposed by Jain & Motwani [17] and Pereira A et al. [18].

Jain and Motwani conducted the study on 20 cadavers and postulated the following classification based on the branching pattern of SMA. She categorized it into the following groups. Group I - normal branching pattern as described in the textbook; Group II - common trunk dividing into the RCA and ICA; Group III - common trunk dividing into the left colic artery and accessory splenic artery [17].

**Figure 4:**
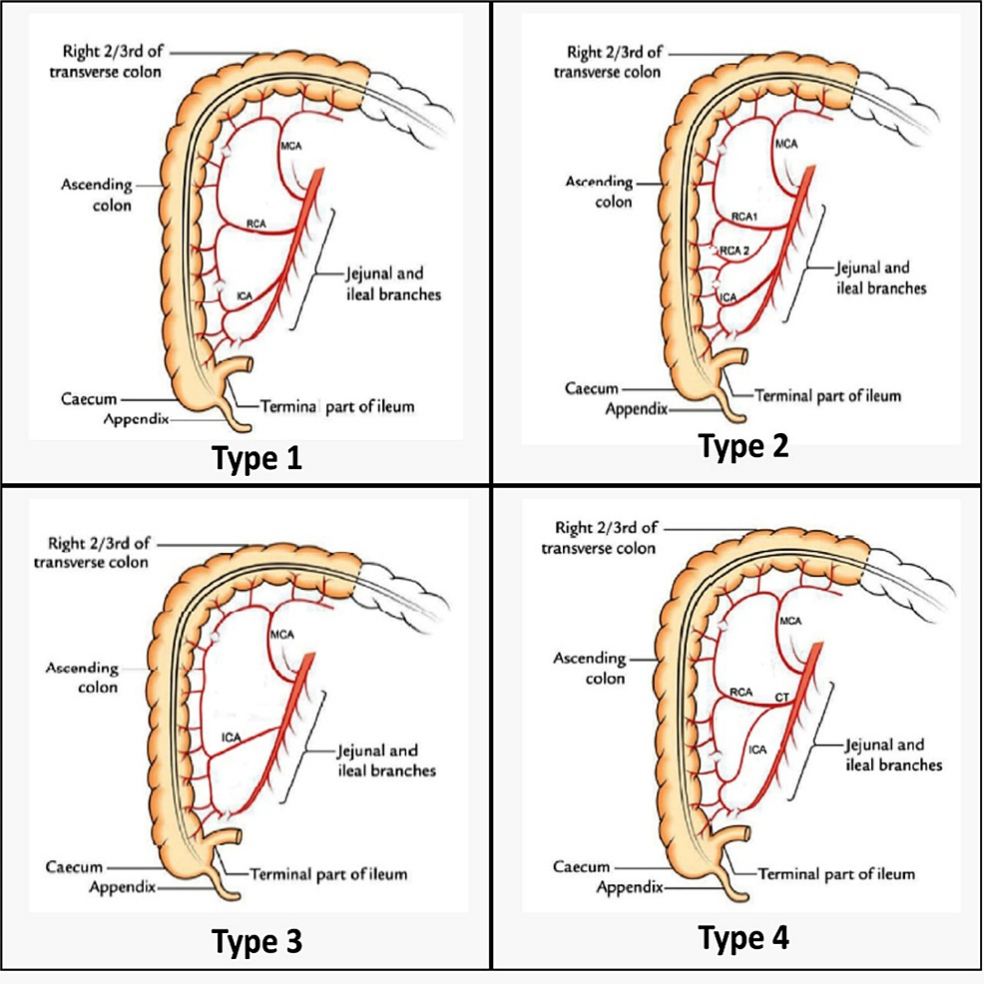
Schematic representation of branching pattern of Superior Mesenteric Artery as per Pereira et al classification The image is our own original creation

Another classification was proposed by Pereira A et al. wherein five types of branching patterns of SMA were described: Type 1 - ascending and transverse colon irrigated by three branches as the middle colic artery, right colic artery, and ileocolic artery; Type 2 - four branches, being one middle colic artery and two right colic arteries and one ileocolic artery; Type 3 - two branches, being one middle colic and one ileocolic artery with the absence of the right colic artery; Type 4 - two main branches, middle colic artery (MCA), and the common trunk of the right colic artery (RCA) and the ileocolic artery (ICA); Type 5 - other than the above four variations [18]. See Figure 4.

Vascular variation in the current case report conforms to Group II of Jain & Motwani and Type 4 of the Pereira A et al. classification. The embryological basis of this vascular variation could possibly relate to errors involving the development of ventral splanchnic arteries. The uniqueness of this current case is highlighted by dual variation involving both positional and vascular variations to the concerned organ. Both simultaneously existing in the same case have not been reported so far.

The clinical presentation of these cases may range from asymptomatic to mimicking acute cholecystitis. Delay in the diagnosis may also lead to a perforated appendix. Because of its proximity to the kidney, suprarenal gland, and liver, it may pose a diagnostic dilemma. A literature review has shown CT to be a more sensitive and specific investigation compared to ultrasound where many cases of the same remained undetected.

## Conclusions

To conclude, a subhepatic cecum and appendix pose a diagnostic dilemma, especially in common cases like appendicitis wherein referred pain may no longer be elicited at Mc Burney's point owing to the positional variation of the cecum and, therefore, the appendix. It can be life-threatening if diagnosed late. It can also co-exist with vascular variation as observed in the study. Vascular variations can lead to iatrogenies during laparotomy. Hence, we strongly recommend a CT angiogram in addition to routine investigation keeping in mind the dual nature of the positional and vascular variation observed in the present cases.
